# Dermatological impact of hand hygiene practices during COVID‐19: A cross‐sectional web‐based survey among doctors in a tertiary care hospital in Eastern India

**DOI:** 10.1111/jocd.15508

**Published:** 2022-12-01

**Authors:** Gaurav Dash, Nibedita Patro, Binayak Chandra Dwari, Kumar Abhishek

**Affiliations:** ^1^ Department of Dermatology Hitech hospital Bhubaneswar India

**Keywords:** COVID‐19, doctors, hand hygiene practices, hand sanitizer, hand washing

## Abstract

**Background:**

The COVID‐19 pandemic continues to persist throughout the world with intermittent exacerbation. The changing trend of hand hygiene practices during this pandemic has led to new onset or aggravation of pre‐existing hand eczema, especially among doctors. There is a paucity of studies regarding skin changes seen with changing hand hygiene practices in the Indian subcontinent.

**Objectives:**

To estimate the frequency of various cutaneous manifestations and associated factors with hand hygiene practices in doctors during COVID‐19 via a web‐based online questionnaire survey.

**Methods:**

It was a cross‐sectional web‐based survey conducted at a tertiary care teaching institute from July 2021 to September 2021. Those doctors (faculty, residents, and interns) of the hospital completing the questionnaire with electronic informed consent were included in the study.

**Results:**

A total of 143 doctors completed the survey. The most common symptoms were dryness in 60 (42%) and itching in 25 (17.5%) doctors. The most common skin changes were scaling in 30 (21%) and redness in 16 (11.2%) doctors. There was a significant association between skin changes and frequency of hand washing and hand sanitizer use (*p* value < 0.05).

**Conclusion:**

The prevalence of hand changes and symptoms was 77% in doctors in our study. The most commonly seen hand changes were scaling followed by redness and symptoms observed were dryness and itching.

## 1 INTRODUCTION

Despite extensive vaccination drive, the COVID‐19 pandemic continues to exist in intermittent waves throughout the world.^1^ Amidst this, hand hygiene practices in the form of hand washing and sanitizer usage are still recommended by world health organization (WHO) as one of the practical options for curbing the transmission of COVID‐19. The Centre for Disease Control and Prevention (CDC) also recommends frequent hand washing and sanitizers like 60% ethanol or 70% isopropanol for 20 s for proper protection against COVID‐19.^2^ The frequency of hand washing and usage of sanitizer has significantly increased in the last 2 years, as seen in various studies.^3^, ^4^, ^5^ It is prominent, especially among doctors being exposed to infection more frequently. This changing trend has led to new onset or aggravation of pre‐existing hand eczema due to disruption of the protective epidermal barrier.^5^ There is gradual depletion of the lipid portion in the natural lipid mantle of skin on contact with lipid emulsifiers like detergents, harsh soaps, and alcohol containing sanitizers. As a result, there is deeper entry of various allergens leading to hand dermatitis (contact dermatitis).^6^, ^7^ Recent studies have found an increased prevalence of such cases among health care workers (HCWs) like doctors involved in the management of COVID‐19.^5^, ^6^, ^7^, ^8^ Cutaneous manifestations are in the form of dryness, itching in milder cases, and blisters, fissures, and bleeding in severe cases.^9^ To our knowledge, there are limited studies regarding skin changes seen with hand hygiene practices in the Indian subcontinent during COVID‐19. This study aimed to enumerate the cutaneous manifestations and associated factors with hand hygiene practices in doctors during COVID.

## 2 MATERIALS AND METHODS

A cross‐sectional web‐based survey was conducted from July 2021 to September 2021 at a tertiary care teaching institute. The doctors (interns, residents, and faculty) working in the institute were invited to participate in the study. The collection of information was done in an online questionnaire format as provided in Appendix S1. Those completing the questionnaire with electronic informed consent were included in the study. The study was approved by the institutional ethical committee with approval no. HMCH/IEC/2021/105 dated 03/07/2021. Statistical analysis was performed using SPSS, version 17.0 software. *p* < 0.05 was considered significant.

## 3 RESULTS

A total of 143 participants, age ranging from 22 to 68 years (mean 31.5 ± 7.2), completed the survey. The male (84) to female (59) ratio was 1.4: 1. Most of the doctors (93) belonged to the age group of 25–35 years (65%).

### 3.1 Medical setup of working

The most common working setup of doctors participating in the survey was the out‐patient department (OPD) in 111 (77.6%), followed by the in‐patient department (IPD) in 78 (54.5%) and casualty in 34 (23.8%) doctors, respectively. (Table 1).

**TABLE 1:** Medical setup

Medical setup	Number	Percentage (%)
OPD (Out‐patient department)	111	77.6
IPD (In‐patient department)	78	54.5
Casualty	34	23.8
ICU (Intensive care unit)	30	21
OT(Operation theater)	29	20.3

There was no significant association between skin changes and different medical setup (*p* Value = 0.689).

### 3.2 Frequency of hand washing per day

Most of the doctors [95 (66.4%)] were in the habit of hand washing more than five times/day, followed by 2–5 times/day in 46 (32.1%).

A significant association was found between skin changes and frequency of hand washing (*p* value = 0.039; Table 2).

**TABLE 2:** Association of skin changes with the frequency of hand washing

Hand washing/day	Skin changes		Total
	Yes	No	
<2 times	1	1	2
2–5 times	28	18	46
>5 times	60	35	95
Total	89	54	143

*Note*: *p* Value = 0.039.

### 3.3 Frequency of hand sanitizer use

The frequency of hand sanitization was more than ten times/day in 67 (46.9%), followed by 5–10 times in 51 (35.7%) doctors, respectively.

A significant association was found between skin changes and frequency of hand sanitizer use (*p* value = 0.012; Table 3).

**TABLE 3:** Association of skin changes with the frequency of hand sanitization

Hand sanitization/day	Skin changes		Total
	Yes	No	
<5 times	9	16	25
5–10 times	35	16	51
>10 times	45	22	67
Total	89	54	143

*Note*: *p* value = 0.012.

### 3.4 Type of hand sanitizer used

The type of hand sanitizer used could not be significantly associated with skin changes (*p* = 0.829). Most of the doctors (60.8%) were using both types of hand sanitizer (aqueous and gel), whereas 36.7% of doctors used only aqueous hand sanitizer.

### 3.5 Skin changes and symptoms in the hands

The most common symptoms were dryness in 60 (42%), followed by itching in 25 (17.5%) and burning in 13 (9.1%) doctors, respectively (Table 4). The most common skin changes were scaling in 30 (21%), followed by redness in 16 (11.2%) and cracking in 14 (9.8%) doctors, respectively (Table 5). There were no skin changes and symptoms in 33 (23%) doctors. History of atopy was seen in 10 (7%) and allergy in 16 (11.2%) doctors, respectively.

**TABLE 4:** Cutaneous symptoms observed during the study

Skin symptoms	Number	Percentage (%)
Dryness	60	42
Itching	25	17.5
Burning	13	9.1
Pain	4	2.8

**TABLE 5:** Cutaneous changes observed during the study

Skin changes	Number	Percentage (%)
Scaling	30	21
Redness	16	11.2
Cracking	14	9.8
Nail fold swelling	7	4.9
Oozing	2	1.4

These skin changes were affecting clinical/nonclinical work in only 10.5% of doctors, but there was no effect in 89.5% of doctors as per the survey.

The common site of involvement was found to be palm (44.8%) followed by dorsa of hand (23.1%) webspace (20.3%) and fingers (18.2%).

### 3.6 Relief of skin changes and symptoms

Most doctors used moisturizers (56.6%) to relieve skin changes and symptoms.

## 4 DISCUSSION

Hand hygiene practices (hand washing and sanitizer use) remain the critical step apart from social distancing and wearing face masks in preventing transmission, thus attenuating the waves of the COVID‐19 pandemic.

A web‐based questionnaire survey was conducted among doctors working in our institution to identify the various skin changes secondary to an increased hand hygiene practices being followed during COVID pandemic. The mean age of doctors participating in the survey was found to be 31.5 years, comparable with the study by Leelawadee et al.,^10^ where it was 32 years. In our study, males (58.7%) outnumbered females (41.3%) in contrast to previous studies by Leelawadee et al.,^10^ Guertler et al.,^11^ Zahr Allayali et al.,^12^ and Keily et al.^13^ where there was female predominance due to inclusion of female nurses as HCWs in most of the studies.

The most common working setup of participating doctors in our survey was the out‐patient department (OPD) in 111 (77.6%), followed by the in‐patient department (IPD) in 78 (54.5%) doctors, respectively. In contrast, medical ward/IPD (41%) was the most common setup in the study by Keily et al.^13^

WHO and the CDC guidelines suggest handwashing for at least 20 s.^14^

It is preferred over alcohol‐based sanitization (60% ethanol or 70% isopropyl alcohol as recommended by CDC), especially when there are visible dirty/greasy hands. It removes the virus mechanically rather than inactivating it.^15^ But sanitizer should be encouraged when hands are not visibly dirty. These hand hygiene practices had reached epic proportions during COVID‐19 pandemic, thus leading to various adverse effects in the form of dryness, scaling, itching and redness in the hand. These are even more exaggerated among HCWs, especially doctors, as they must strictly comply with these hand hygiene practices more frequently and for a prolonged period to avoid infection and thus transmission.^16^, ^17^

The most common symptom in our survey was dryness [60 (42%)], followed by itching [25 (17.5%)]. It is similar to the study by Keily et al.,^13^ Leelawadee et al.,^10^ Zahr Allayali et al.,^12^ Guertler et al.,^11^ and Agarwal et al.^18^ It is secondary to depletion of the lipid barrier due to lipid‐emulsifying detergents and lipid‐dissolving alcohols.^18^ The most common skin changes seen were scaling in 30 (21%) and redness in 16 (11.2%) doctors, respectively. In contrast, erythema followed by scaling were seen in study by Reinholz et al.,^19^ Zahr Allayali et al.,^12^ and Guertler et al.^11^ In our study, 33(23%) doctors did not complain of any skin symptoms or signs. History of atopy was seen in 10 (7%) doctors in our study, similar to study by Guertler et al.^11^ and Reinholz et al.^19^

Skin changes in our study were significantly associated with the frequency of hand washing. The findings substantiate with the study by Leelawadee et al.^10^ where the risk of hand eczema was enhanced by increased frequency of handwashing (more than 10 times/day). Frequent handwashing for a prolonged period causes swelling, denaturation of stratum corneum and decreased attachment between corneocytes leading to epidermal barrier disruption. Consequently, there is increased skin sensitivity to various allergens, thus culminating to contact dermatitis.^20^ It is even more exaggerated in people with pre‐existing hand eczema and atopic dermatitis.

Barrier repair hand creams replenish the lost skin lipids and form a protective layer over the epidermis, thus improving the skin's barrier function and preventing hand hygiene‐related hand eczema.^21^ There was a significant reduction in incidences of hand eczema among healthcare workers with proper regular use of hand creams, as observed in a randomized controlled trial.^15^ Most doctors in our study used moisturizers (56.6%) to relieve skin changes and symptoms. It is comparable to study by Reinholz et al.^19^ where 77.3% of employees who developed eczema used emollients for treatment.

Multiple factors have led to hand eczema in healthcare workers. These include increased frequency of hand washing or sanitization, exposure to soaps, detergents, prolonged usage of gloves, atopic dermatitis, and infrequent application of moisturizers.^22^ Many guidelines have been laid down for the prevention of hand dermatitis. Use of lukewarm water, fragrance and preservative‐free soap followed by soft pat drying is encouraged.^20^ Liberal use of hypoallergenic fragrance‐free lipid‐rich moisturizers containing mineral oil or petrolatum immediately after hand wash is an essential step in addition to the above.^23^ In contrast, it should be applied after using hand sanitizer once they are dry. Alcohol‐based hand sanitizer should only be encouraged when hands are not visibly dirty because of less irritant potential. Glycerol containing hand sanitizers are also available for their moisturizing effect. Most cases of hand eczema improve with topical corticosteroids and emollients. Dermatologist consultation is necessary only in very severe cases which require systemic steroids and immunosuppressive agents such as methotrexate and cyclosporine.^23^

## 5 LIMITATIONS

There is recruitment of more younger technology savvy population coincidentally in these web‐based questionnaire survey. So, the prevalence found in our study may not reflect the exact scenario seen in the general population of doctors. Larger multicentric studies need to be performed for elaborate data.

## 6 CONCLUSION

Excess use of hand hygiene practices in the form of hand sanitizer and handwashing has increased the frequency of hand eczema in healthcare workers, especially doctors. The prevalence of hand changes and symptoms was 77% in our study. The most commonly seen hand changes were scaling followed by redness. The most frequently observed hand symptoms were dryness and itching. The risk factors were frequency, type of hand sanitizer, frequency of handwashing per day. There was a significant reduction in incidences of hand eczema among doctors with proper regular use of moisturizers. The knowledge of various skin changes associated with frequent hand hygiene practices will help in taking necessary preliminary steps to prevent the same leading to unhindered duty performance of doctors.

## ETHICAL APPROVAL

The authors confirm that the ethical policies of the journal has been adhered to as per the journal guidelines. The medical ethics body of Hi‐Tech Medical College and Hospital, Bhubaneswar, India has approved the study vide letter no ‐ HMCH/IEC/2021/105, dated 03.07.2021. All the subjects signed the written consent form.

## Supporting information


**Appendix S1:** Supporting information

## Data Availability

The data that support the findings of this study are available from the corresponding author upon reasonable request.
