# Supplementary material for: Dermatological impact of hand hygiene practices during COVID‐19: A cross‐sectional web‐based survey among doctors in a tertiary care hospital in Eastern India
Source: J Cosmet Dermatol. 2022 Dec 1;22(1):21–5. doi: 10.1111/jocd.15508 (PMC9878225; doi:10.1111/jocd.15508)
Supplement: Supplementary file 1 — Appendix S1: Supporting information [file JOCD-22-21-s001.docx]

|  |
| --- |
|  |
| Questionnaire |
|  |

1. I agree to be a part of this study conducted at Hitech Medical College & Hospital, Bhubaneswar

Yes

No

1. Age in years

3. Gender

1. Male
2. Female

4.Any history of

a) Atopy

b) Allergy

c)None

5. Which setup you are working?

1. OPD
2. IPD
3. Casualty
4. ICU
5. OT
6. Others

6. Frequency of handwashing per day

1. > 5
2. 2-5
3. < 2

7. Type of Sanitiser used

1. Gel
2. Aqueous
3. both

8. How frequently you use hand sanitizer

1. > 10
2. 5-10
3. < 5

9. Dermatological changes seen

1. Itching
2. burning
3. Redness
4. Burning
5. Pain
6. Scaling
7. Oozing
8. Cracking
9. Nail fold swelling
10. Dryness
11. None
12. Other

10. Does it affect your clinical or non-clinical work

Yes

No

11. Site of involvement

1. Palm
2. Dorsa of hand
3. Webspace
4. Fingers
5. Nail folds
6. None
7. Other

12. How did you get relief

1. Moisturiser
2. Topical steroid
3. Oral steroids
4. Topical antifungal
5. Oral antifungal;
6. Other
